# Obese patients with higher TSH levels had an obvious metabolic improvement after bariatric surgery

**DOI:** 10.1530/EC-21-0360

**Published:** 2021-09-15

**Authors:** Nannan Bian, Xiaomeng Sun, Biao Zhou, Lin Zhang, Qiu Wang, Yu An, Xiaohui Li, Yinhui Li, Jia Liu, Hua Meng, Guang Wang

**Affiliations:** 1 Department of Endocrinology, Beijing Chao-Yang Hospital, Capital Medical University, Beijing, China; 2 Departments of General Surgery and Obesity and Metabolic Disease Center, China-Japan Friendship Hospital, Beijing, China

**Keywords:** obesity, bariatric surgery, adipose tissue insulin resistance, thyroid-stimulating hormone

## Abstract

**Objective:**

Bariatric surgery has become the most effective treatment for morbid obesity. Increasing evidence showed that bariatric surgery can alleviate insulin resistance and influence thyroid function. This study aimed to investigate the relationship between changes in thyroid function and adipose tissue insulin resistance (adipo-IR) after bariatric surgery.

**Methods:**

A total of 287 non-diabetic participants with regular thyroid function were recruited and divided into the lean, overweight and obese groups. Among them, 50 morbidly obese patients submitted to bariatric surgery.

**Results:**

The obese group had a higher level of adipo-IR, thyroid-stimulating hormone (TSH), free triiodothyronine (FT3), FT3/free thyroxine (FT4) and metabolism disorders than the lean and overweight groups. BMI was correlated with TSH, FT3, FT3/FT4 and adipo-IR (*r* = 0.309, 0.315, 0.322 and 0.651, respectively, all *P* < 0.001). Adipo-IR was significantly correlated with TSH (*r* = 0.402, *P* < 0.001), FT3 (*r* = 0.309, *P* < 0.001), and FT3/FT4 (*r* = 0.228, *P* < 0.05). Bariatric surgery resulted in a sharp decline in BMI, adipo-IR, TSH, FT3 and FT3/FT4 levels, meanwhile, metabolic disorders improved. The decrease in BMI after bariatric surgery was significantly correlated with reductions in adipo-IR (*r* = 0.577, *P* < 0.001) and TSH (*r* = 0.401, *P* = 0.005). Interestingly, the fasting blood glucose, fasting insulin, adipo-IR and TSH in the higher TSH group decreased more remarkably than in the lower TSH group.

**Conclusion:**

Obese individuals with higher TSH levels had an obvious metabolic improvement after bariatric surgery.

## Introduction

The morbidity of overweight and obesity has increased globally (1). During 2013–2014 in the United States, obesity was 40.4% among women and 35% among men. The corresponding incidences for morbid obesity were 9.9% for women and 5.5% for men (2). Obesity is an independent risk factor for insulin resistance (IR), metabolic syndrome and type 2 diabetes (T2D). For morbidly obese patients, lifestyle interventions and medication fail to reduce body weight durably, however, bariatric surgery is the most effective way to lose weight and exert beneficial effects on metabolism (3). Adipose tissue insulin resistance (adipo-IR) is one of the pathophysiological characteristics of obesity (4, 5, 6). Hepatic and muscular IR have received extensive attention and been widely researched, but few studies have focused on adipo-IR.

Thyroid hormones play a core part in energy metabolism and body weight balance (7). Mild deviation of thyroid function within the reference range has been observed in obese individuals, such as elevated serum thyroid-stimulating hormone (TSH) level and high transformation of thyroxine (T4) to triiodothyronine (T3) (8, 9, 10). Serum TSH and free triiodothyronine (FT3) levels decreased in obese individuals after bariatric surgery (11). However, to our knowledge, there have been no studies on the association between thyroid function (TSH, FT3, free thyroxine (FT4) and FT3/FT4) and adipo-IR. Our study investigated the relationship between thyroid function and adipo-IR in non-diabetic obese patients with euthyroid. Then we observed the change of metabolism and the relationship between the change of thyroid function and adipo-IR in morbidly obese patients 6 months after bariatric surgery.

## Materials and methods

### Study design and study population

This study is a retrospective study. A total of 287 non-diabetic patients with regular thyroid function were recruited from the Endocrinology Department of Beijing Chao-Yang Hospital affiliated with Capital Medical University and Obesity and Metabolic Disease Center of China–Japan Friendship Hospital affiliated with Capital Medical University from January 2017 to December 2020. Thyroid function, fasting free fatty acids (FFA), fasting blood glucose (FBG) and fasting insulin (FINS) were measured in these patients. Individuals with diabetes, thyroid dysfunction, secondary obesity due to pregnancy or possible pregnancy, endocrine disorders, hepatic and renal impairment, cardiocerebral vascular disease, infectious disease, systemic inflammatory disease, mental illnesses, cancer or severe hereditary diseases were excluded. No subject took hypoglycemic drugs, antithyroid and levothyroxine drugs.

We first divided the 287 participants into 3 groups, 76 lean individuals with BMI < 25 kg/m^2^, 64 overweight individuals with BMI 25.0–29.9 kg/m^2^ and 147 individuals with BMI ≥ 30.0 kg/m^2^. The baseline characteristics, glucose and lipid metabolism index, insulin secretory index, adipo-IR index and thyroid function were compared among the three groups. Then we analyzed the correlations among BMI, adipo-IR and thyroid function.

We further investigated 50 morbidly obese patients who had undergone bariatric surgery and performed a follow-up visit at 6 months. We compared the changes of BMI, glucose and lipid metabolism, insulin secretory, adipo-IR and thyroid function before and 6 months after bariatric surgery and observed the relationships among changes of BMI, adipo-IR and thyroid function. In order to further observe the effects of lower and higher TSH levels within the regular range on blood glucose, lipid and thyroid function index, we divided the 50 participants into the lower TSH group and the higher TSH group.

We confirm that the work was conducted in accordance with the Declaration of Helsinki (1964). This study was approved by the Ethics Committee of the China–Japan Friendship Hospital (2019-103-K71) and the Ethics Committee of the Beijing Chao-Yang Hospital, Capital Medical University. All enrolled participants provided written informed consent.

### Clinical and biochemical measurements

All participants underwent anthropometric tests and laboratory assays. Body weight and height were measured by the same trained team to approximately 0.1 kg and 0.1 cm, respectively. Venous blood samples were collected after overnight fasting for 10 to 12 h. Oral glucose tolerance test (OGTT), a 75 g oral glucose solution, was used to assess insulin sensitivity and insulin secretion function in the morbidly obese patients at baseline and 6 months after bariatric surgery. Plasma glucose and insulin concentrations were tested at 0, 30, 60, 120 and 180 min relative to glucose ingestion.

Triglyceride (TG), total cholesterol (TC), high-density lipoprotein cholesterol (HDL-C) and low-density lipoprotein cholesterol (LDL-C) levels were determined by colorimetric enzymatic assays using an autoanalyzer (Hitachi 7170). FBG was measured by the glucose oxidase method, and FINS was measured by the chemiluminescence method. The FFA concentration was quantified using the enzymatic colorimetric method. TSH, FT4 and FT3 were measured by electrochemiluminescence immunoassay using an Abbott Architect i2000 (Abbott Diagnostics).

BMI was calculated as the weight (kg) divided by the height (m^2^). IR was evaluated with the homeostasis model assessment of insulin resistance (HOMA-IR) according to the following formula: HOMA-IR = (FBG (mmol/L) × FINS (μIU/mL)/22.5) (12, 13). Central resistance to thyroid hormones was evaluated with the thyrotroph T4 resistance index (TT4RI). TT4RI was calculated as FT4 (pmol/L)×TSH (mIU/L) (14). Adipo-IR was calculated as fasting FFA concentration (mmol/L) × FINS (pmol/L). The trapezoidal method was used to calculate the areas under the curve (AUC) for glucose and insulin. Early phase insulin secretion index (EISI) was measured according to the following formula: ΔI30/ΔG30 = (INS30 min – FINS)/(Glu30 min − FBG). Disposition index (DI) was used to adjust insulin secretion for the extent of IR and was calculated as ΔI30/ΔG30/HOMA-IR.

### Statistical analysis

Normally distributed Continuous variables were expressed as mean and s.d., while skewed distribution variables were shown as median and interquartile range. The differences among multiple groups were analyzed by Kruskal–Wallis test or ANOVA test. Comparison between the two groups was analyzed using the independent Student’s *t*-test or Mann–Whitney *U-*test. Bonferroni corrections were applied for multiple comparison correction. The proportions were analyzed by χ^2^ tests. Paired sample *t*-test and Wilcoxon signed-rank test were used to compare preoperative and postoperative levels of relevant indicators. Bivariate correlation analyses (Spearman’s test and Pearson’s test) were used to evaluate relationships between variables. A two-tailed *P* value < 0.05 was considered significant, and issues of multiple testing were taken into consideration by considering *P* values adjusted for the false discovery rate (FDR). Data processing was done with SPSS 26.0 (SPSS, Inc.), GraphPad Prism 8.0 (Graphpad Software Inc.) and R version 4.1.1.

## Results

### Clinical characteristics of the lean, overweight and obese groups

Baseline characteristics of the lean, overweight and obese groups are summarized in Table 1. Of the 287 participants in this study, 76 subjects were lean, 64 were overweight, and 147 were obese. There were no statistical differences among these three groups in dyslipidemia, CHOL and FT4. However, there were significant differences in other anthropometric and laboratory tests, including age, gender, BMI, TG, CHOL, HDL-C, LDL-C, A1C, FBG, FINS, fasting FFA, HOMA-IR, adipo-IR, TSH, FT3, FT3/FT4 and TT4RI. From lean to obesity, patients showed metabolism disorders, and adipo-IR, TSH, FT3, FT3/FT4 and TT4RI significantly increased.

**Table 1:** Comparisons of basic characteristics among lean, overweight and obese people. Data are shown as median and upper and lower quartiles unless indicated otherwise. LDLC and FT3 are shown as means ± s.d.

	Lean, *n* = 76	Overweight, *n* = 64	Obesity, *n* = 147	*P* value
Age (years)	44.0 (35.0–60.3)	44.5 (31.0–53.8)	32.0 (25.0–37.0)^a,b^	0.000
Gender (female)	63 (82.9%)	37 (57.8%)^a^	91 (61.9%)^a^	0.003
BMI (kg/m^2^)	22.4 (21.3–23.4)	27.7 (26.2–29.0)^a^	38.2 (34.1–43.0)^a,b^	0.000
Dyslipidemia	25 (32.9%)	21 (32.8%)	64 (43.5%)	0.187
Triglyceride (mmol/L)	1.09 (0.82–1.52)	1.54 (1.14–2.22)	1.62 (1.23–2.12)^c^	0.000
TC (mmol/L)	4.43 (3.89–5.33)	4.46 (4.05–5.19)	4.64 (4.09–5.36)	0.435
HDL-C (mmol/L)	1.45 (1.10–1.67)	1.10 (0.94–1.32)^a^	1.00 (0.87–1.15)^a,b^	0.000
LDL-C (mmol/L)	2.67 ± 1.00	2.78 ± 0.79	3.09 ± 0.78^a,d^	0.001
AIC (%)	5.60 (5.30–6.00)	5.90 (5.50–6.30)^c^	5.80 (5.43–6.78)^c^	0.013
FBG (mmol/L)	4.88 (4.64–5.12)	5.13 (4.82–6.25)^a^	5.77 (4.96–6.72)^a^	0.000
FINS (mIU/L)	7.4 (5.0–10.1)	11.7 (8.7–18.3)^a^	27.8 (18.0–43.3)^a,b^	0.000
Fasting FFA (mmol/L)	0.51 (0.42–0.71)	0.55 (0.40–0.71)	0.64 (0.50–0.84)^a,b^	0.001
HOMA-IR	1.61 (1.07–2.32)	3.09 (2.08–5.96)^a^	7.65 (4.14–11.30)^a,b^	0.000
Adipo-IR (mmol/L × pmol/L)	26 (18–38)	56 (30–74)^a^	125 (71–198)^a,b^	0.000
TSH (μIU/mL)	1.93 (1.35–2.39)	2.07 (1.30–2.68)	2.41 (1.72–3.10)^a,d^	0.000
FT3 (pmol/L)	4.65 ± 0.48	4.82 ± 0.59	5.11 ± 0.63^a,d^	0.000
FT4 (pmol/L)	16.4 (15.5–17.9)	15.3 (14.0–17.4)	16.1 (14.3–17.4)	0.079
FT3/FT4	0.27 (0.26–0.30)	0.31 (0.12–0.33)^a^	0.31 (0.29–0.34)^a^	0.000
TT4RI	31.9 ± 11.3	32.3 ± 13.1^c^	38.6 ± 14.6^b,c^	0.001

^a^Significantly different at *P* < 0.01 vs the lean group; ^b^Significantly different at *P* < 0.01 vs the overweight group; ^c^Significantly different at *P* < 0.05 vs the lean group; ^d^Significantly different at *P* < 0.05 vs the overweight group.

adipo-IR, adipose tissue insulin resistance; FBG, fasting blood glucose; FFA, free fatty acid; FINS, fasting insulin; FT3, free triiodothyronine; FT4, free thyroxine; HDL-C, high-density lipoprotein cholesterol; HOMA-IR, homeostasis model assessment of insulin resistance; LDL-C, low-density lipoprotein cholesterol; TC, total cholesterol; TSH, thyroid-stimulating hormone; TT4RI, thyrotroph T4 resistance index.

### Relationships among BMI, TSH, FT3, FT3/FT4 and adipo-IR in non-diabetic patients

**Figure 1:**
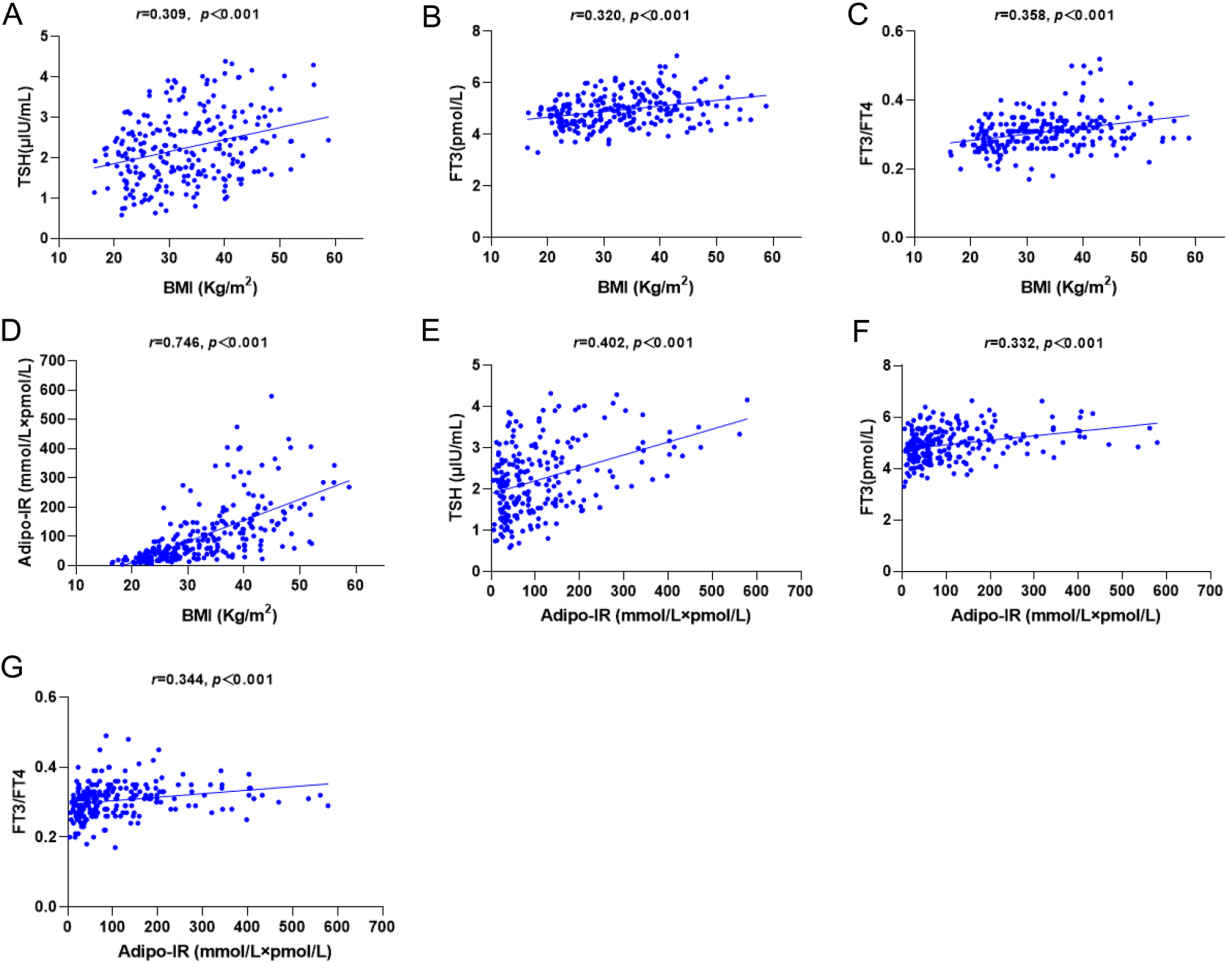
Relationships among BMI, TSH, FT3, FT3/FT4 and adipo-IR in non-diabetic patients. (A) BMI was positively correlated with TSH. (B) BMI was positively correlated with FT3. (C) BMI was positively correlated with FT3/FT4. (D) BMI was positively associated with Adipo-IR. (E) Adipo-IR was positively associated with TSH. (F) Adipo-IR was positively correlated with FT3. (G) Adipo-IR was positively correlated with FT3/FT4.

Correlation between BMI, adipo-IR, TSH and clinical parameters are shown in Supplementary Table 1. BMI was linearly correlated with TSH, FT3, FT3/FT4 and adipo-IR (TSH: *r* = 0.309; FT3: *r* = 0.320; FT3/FT4: *r* = 0.358; adipo-IR:* r* = 0.746, all *P* < 0.001; Fig. 1A, B, C and D). Interestingly, adipo-IR was correlated with TSH (*r* = 0.402, *P* < 0.001), FT3 (*r* = 0.332, *P* < 0.001), and FT3/FT4 (*r* = 0.344, *P* < 0.000; Fig. 1E, F and G).

### Baseline characteristics and comparison of biochemical, thyroid function and adipo-IR before and 6 months after bariatric surgery

Clinical and biochemical characteristics of 50 (18 men, 32 women; mean age was 27.6 ± 6.2 years) morbidly obese patients before and 6 months after bariatric surgery are given in Table 2. The change of CHOL, LDL-C and FT4 had no statistical differences. However, BMI, dyslipidemia, TG, A1C, FBG, FINS, fasting FFA, HOMA-IR, adipo-IR, TSH, FT3, FT3/FT4 and TT4RI levels were significantly declined, HDL-C level was significantly elevated 6 months after surgery.

**Table 2:** Anthropometric, metabolic and thyroid function in non-diabetic obese patients before and 6 months after bariatric surgery. Data are shown as median and upper and lower quartiles unless indicated otherwise. BMI, HDL-C, fasting glucose, fasting insulin, TSH, FT3 and FT4 are shown as means ± s.d.

	Group		Change after surgery	*P*
	Baseline, *n* = 50	6 month after surgery, *n* = 50		
BMI (kg/m^2^)	40.7 ± 6.1	29.3 ± 5.4	−11.4 (−13.4 to −9.6)	0.000
Dyslipidemia	22 (44.0%)	13 (26.0%)	−9 (−18.0%)	0.049
Triglyceride (mmol/L)	1.55 (1.15 to 2.00)	0.95 (0.73 to 1.11)	−0.51 (−1.06 to −0.15)	0.000
TC (mmol/L)	4.68 (4.24 to 5.56)	4.58 (3.84 to 4.98)	−0.15 (−0.71 to 0.36)	0.275
HDL-C (mmol/L)	1.09 ± 0.24	1.20 ± 0.26	0.09 (−0.09 to 0.23)	0.004
LDL-C (mmol/L)	3.08 (2.69 to 3.58)	2.88 (2.39 to 3.34)	−0.20 (−0.59 to 0.28)	0.201
A1C (%)	5.50 (5.40 to 5.80)	5.10 (4.90 to 5.30)	−0.50 (−0.70 to −0.20)	0.000
FBG (mmol/L)	5.63 ± 0.77	4.59 ± 0.54	−0.92 (−1.40 to −0.46)	0.000
FINS (mIU/L)	35.2 ± 16.4	9.9 ± 5.6	−20.8 (−39.1 to −12.9)	0.000
Fasting FFA (mmol/L)	0.76 (0.60 to 0.87)	0.57 (0.44 to 0.78)	−0.17 (−0.38 to 0.08)	0.013
HOMA-IR	7.6 (4.3 to 13.8)	1.9 (1.5 to 3.6)	−5.7 (−10.8 to −2.4)	0.000
Adipo-IR (mmol/L × pmol/L)	141 (99 to 228)	44 (22 to 75)	−102 (−195 to −59)	0.000
TSH (μIU/mL)	2.45 ± 0.85	2.01 ± 0.79	−0.45 (−0.83 to 0.06)	0.000
FT3 (pmol/L)	5.31 ± 0.60	4.47 ± 0.74	−0.80 (−1.26 to −0.39)	0.000
FT4 (pmol/L)	16.4 ± 2.0	16.6 ± 2.3	0.1 (−1.0 to 1.6)	0.269
FT3/FT4	0.32 (0.30 to 0.35)	0.27 (0.25 to 0.28)	−0.05 (−0.08 to −0.03)	0.000
TT4RI	40.8 ± 15.2	33.1 ± 13.0	−6.5 (−14.6 to 1.0)	0.000

adipo-IR, adipose tissue insulin resistance; Fasting FFA, fasting free fatty acid; FBG, fasting blood glucose; FINS, fasting insulin; FT3, free triiodothyronine; FT4, free thyroxine; HDL-C, high-density lipoprotein cholesterol; HOMA-IR, homeostasis model assessment of insulin resistance; LDL-C, low-density lipoprotein cholesterol; TC, total cholesterol; TSH, thyroid-stimulating hormone; TT4RI, thyrotroph T4 resistance index.

### Effects of bariatric surgery on glucose homeostasis and insulin sensitivity in morbidly obese patients

**Figure 2:**
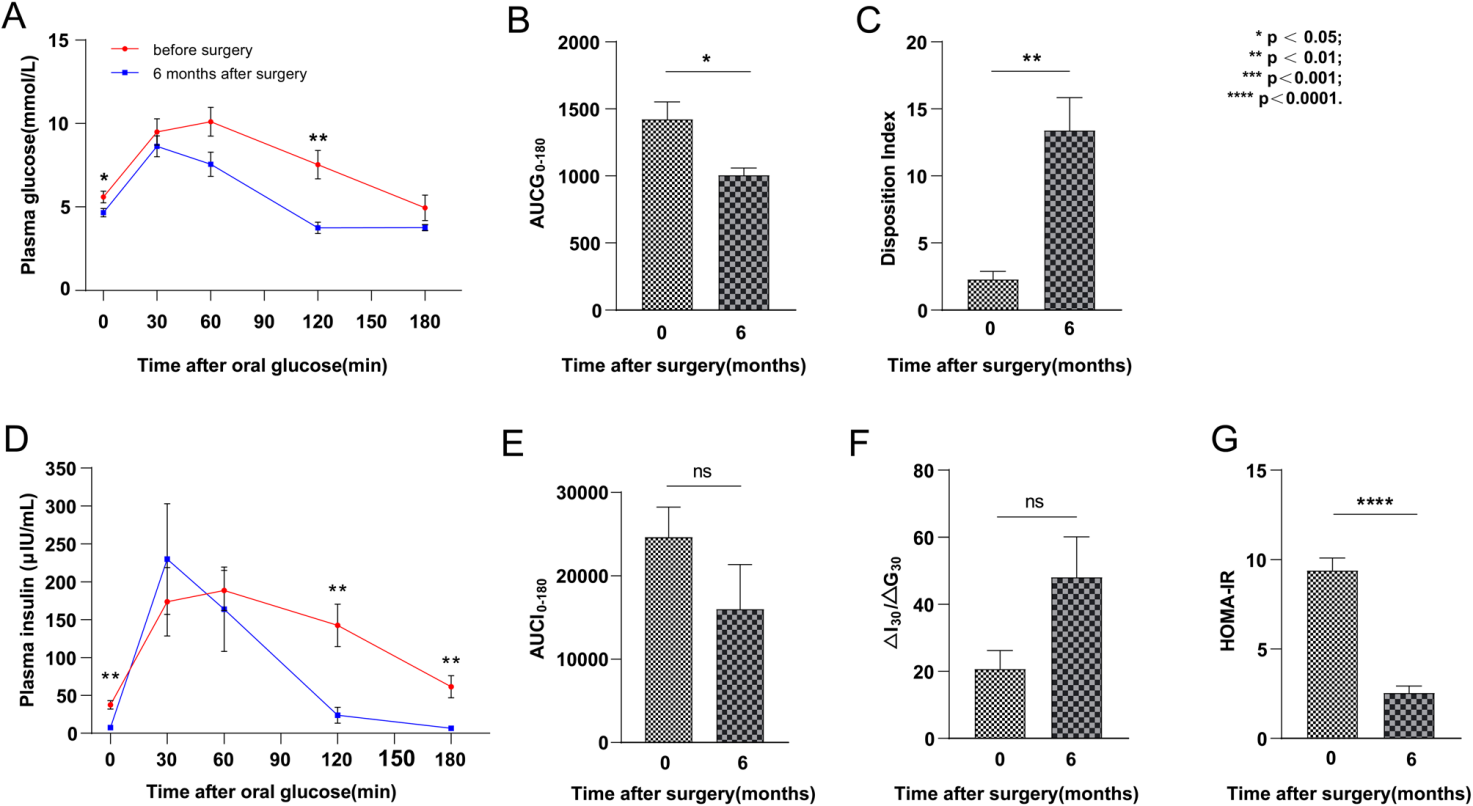
Effects of bariatric surgery on glucose homeostasis and insulin sensitivity in non-diabetic patients. (A) Glucose tolerance improved 6 months after surgery. (B) The AUC of plasma glucose was significantly declined 6 months after surgery. (C) DI was elevated 6 months after surgery. (D) Insulin release was significantly decreased after surgery. (E) There was no difference in AUC of insulin secretion (F) EISI was elevated after surgery but was not significant. (G) HOMA-IR improved after surgery.

OGTT was used to evaluate insulin sensitivity and insulin secretion function among these individuals. Glucose tolerance improved 6 months after surgery (Fig. 2A), and the AUC of plasma glucose was significantly reduced (1423 ± 367 at 0 months vs 1006 ± 152 at 6 months, Fig. 2B). DI was significantly increased (2.3 ± 1.6 at 0 months vs 13.4 ± 6.9 at 6 months, Fig. 2C). Insulin release was significantly declined after surgery (Fig. 2D), and the AUC of insulin release (24,642 ± 10,174 at 0 months vs 16,009 ± 15,143 at 6 months) decreased but had no significance (Fig. 2E). EISI (ΔI_30_/ΔG_30_) was increased 6 months after bariatric surgery but was not statistically significant (*P* = 0.057, Fig. 2F). Bariatric surgery significantly improved HOMA-IR (Fig. 2G). In summary, bariatric surgery notably improved glucose homeostasis and insulin sensitivity.

### Correlations between the decrease in BMI and reductions in adipo-IR and thyroid function

**Figure 3:**
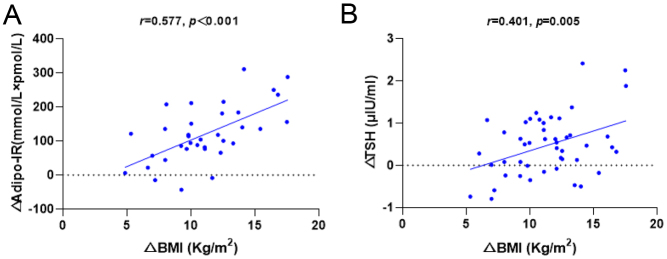
Changes in adipo-IR after bariatric surgery were positively correlated with changes in BMI and TSH. (A) The change of BMI was correlated with the change of Adipo-IR. (B) The change of BMI was associated with the change of TSH.

Correlation between the change in BMI, adipo-IR, TSH and clinical parameters before and after bariatric surgery are presented in Supplementary Table 2. We also observed that the decrease in BMI after bariatric surgery was associated with the decreases in adipo-IR and TSH (adipo-IR: *r* = 0.577, *P* < 0.001; TSH: *r* = 0.401, *P* = 0.005; Fig. 3A and B). However, we did not observe a linear correlation between the decline in BMI and the decrease in FT3 (*r* = 0.065, *P* = 0.662) and FT3/FT4 (*r* = 0.047, *P* = 0.753).

### Comparison of clinical parameters of morbidly obese patients with different TSH levels 6 months after bariatric surgery

**Figure 4:**
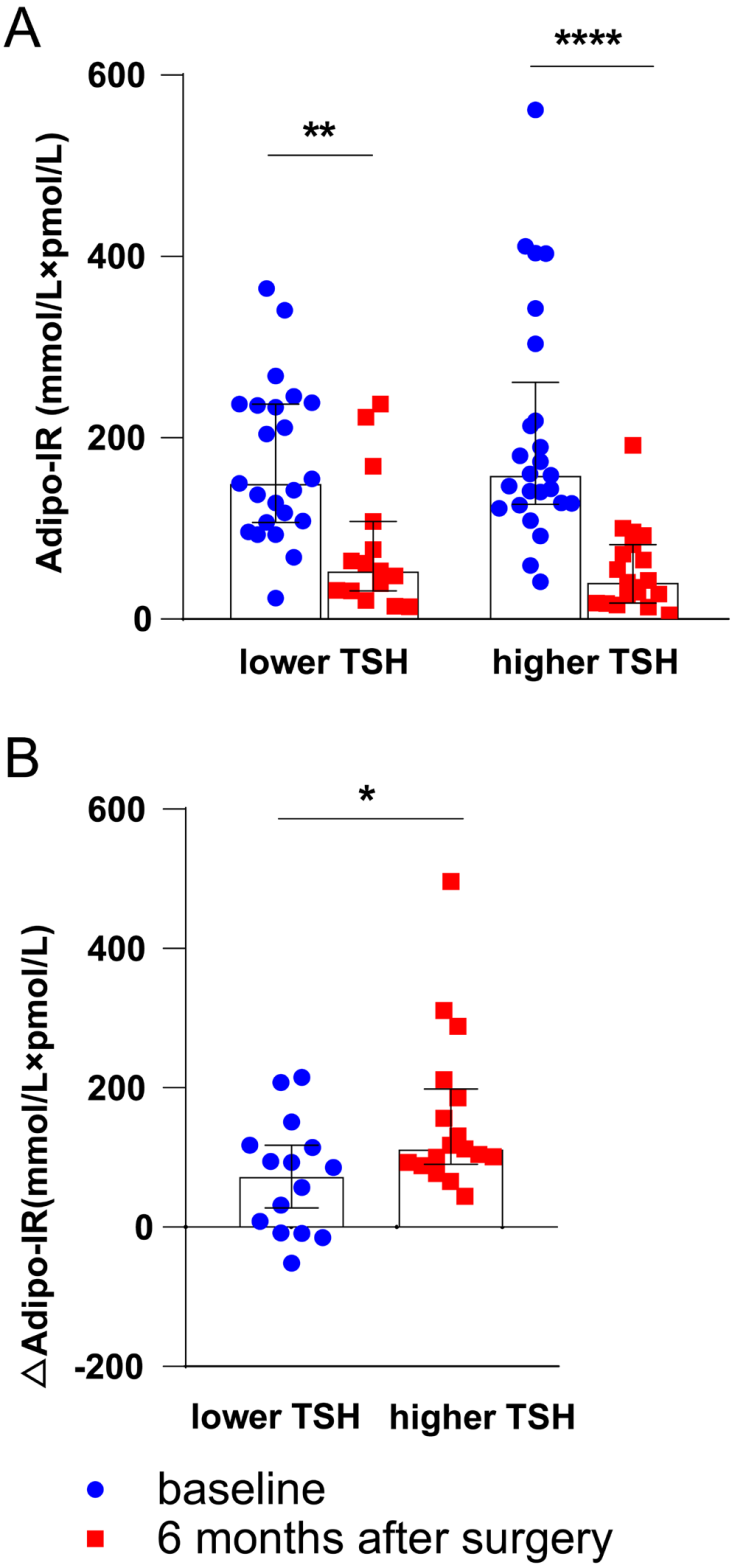
Serum adipo-IR levels of lower TSH group and higher TSH group before and 6 months after bariatric surgery. (A) Adipo-IR level of lower TSH group and higher TSH group at baseline and 6 months after surgery. (B) The change of Adipo-IR in the lower and higher TSH groups.

According to preoperative TSH levels, participants were further divided into a lower TSH group (<2.68 μIU/mL) and a higher TSH group (>2.68 μIU/mL). The changes in BMI, dyslipidemia, triglyceride, CHOL, HDLC, LDLC, A1C, fasting FFA, HOMA-IR, FT3, FT4 and FT3/FT4 levels were same between the lower and higher TSH group 6 months after bariatric surgery (all *P* > 0.05; Table 3). However, the decrease in FBG, FINS, adipo-IR, TSH and TT4RI levels were more obvious in the higher TSH group than in the lower TSH group (all *P* < 0.05; Table 3). Interestingly, 6 months after bariatric surgery, the adipo-IR levels significantly decreased in both groups, but the adipo-IR in the higher TSH group decreased more remarkably than in the lower TSH group (−104 (−186 to −77) vs −86 (−118 to −9), *P* < 0.05; Fig. 4A, B and Table 3).

**Table 3:** Comparison of clinical parameters after bariatric surgery in obese patients with different TSH levels. Data are shown as median and upper and lower quartiles unless indicated otherwise. BMI, HDLC, fasting glucose, fasting insulin, TSH, FT3 and FT4 are shown as means ± s.d.

Parameters	Lower TSH group (*n* = 25)			Higher TSH group (*n* = 25)			*P*
	Baseline	6 month after surgery	Change after surgery	Baseline	6 month after surgery	Change after surgery	
Age (years)	27.0 ± 6.4			28.2 ± 6.1			–
Gender (female,%)	17 (68)			15 (60)			–
BMI (kg/m^2^)	40.5 ± 5.5	29.0 ± 5.2^a^	−11.4 (−13.4 to −9.3)	41.1 ± 6.7	29.5 ± 5.8^a^	−11.6 (−13.6 to −9.7)	0.766
Dyslipidemia (%)	11 (44)	6 (24)	−5 (20)	11 (44)	7 (28)	−4 (16)	0.833
Triglyceride (mmol/L)	1.28 (0.87 to 1.74)	0.94 (0.7 to 1.09)^a^	−0.31 (−0.66 to −0.07)	1.71 (1.21 to 2.16)	1.00 (0.76 to 1.25)^a^	−0.71 (−1.24 to −0.20)	0.144
TC (mmol/L)	4.69 ± 1.01	4.56 ± 1.04	−0.22 (−0.72 to 0.33)	4.92 ± 0.76	4.72 ± 0.79	−0.23 (−0.79 to 0.33)	0.717
HDL-C (mmol/L)	1.09 (0.90 to 1.32)	1.24 (1.11 to 1.32)^b^	0.11 (−0.08 to 0.21)	1.05 (0.91 to 1.17)	1.09 (0.98 to 1.45)^b^	0.10 (−0.05 to 0.23)	0.920
LDL-C (mmol/L)	3.03 ± 0.81	2.89 ± 0.71	−0.19 (−0.58 to 0.21)	3.21 ± 0.56	3.04 ± 0.64	−0.23 (−0.72 to 0.31)	0.776
A1C (%)	5.50 (5.40 to 5.68)	5.05 (4.90 to 5.30)^a^	−0.60 (−0.70 to −0.20)	5.60 (5.40 to 5.88)	5.20 (5.00 to 5.30)^a^	−0.45 (−0.88 to −0.20)	0.573
FBG (mmol/L)	5.41 ± 0.81	4.53 ± 0.58^a^	−0.69 (−1.00 to −0.45)	5.89 ± 0.65	4.63 ± 0.52^a^	−1.13 (−1.75 to −0.64)	0.034
FINS (mIU/L)	27.9 (18.7 to 44.2)	12.6 (6.7 to 23.2)^a^	−8.4 (−20.8 to 0.5)	33.8(26.1 to 53.0)	10.3 (6.8 to 18.1)^a^	−20.5 (−39.1 to −15.4)	0.025
Fasting FFA (mmol/L)	0.77 (0.66 to 0.87)	0.68 (0.52 to 0.81)	−0.10 (−0.36 to 0.08)	0.73 (0.49 to 0.82)	0.48 (0.36 to 0.83)	−0.19 (−0.39 to 0.05)	0.535
HOMA-IR	6.3 (4.2 to 14.3)	1.7 (1.2 to 3.9)^b^	−4.6 (−11.2 to −1.5)	7.6 (5.0 to 17.7)	2.2 (0.9 to 4.8)^a^	−5.2 (−14.0 to −2.1)	0.736
Adipo-IR (mmol/L × pmol/L)	155 (108 to 238)	48 (26 to 92)^b^	−86 (−118 to −9)	159 (127 to 261)	41(18 to 82)^a^	−104 (−186 to −77)	0.044
TSH (μIU/mL)	1.79 ± 0.57	1.66 ± 0.58	−0.14 (−0.53 to 0.38)	3.21 ± 0.38	2.34 ± 0.82^a^	−0.81 (−1.14 to −0.17)	0.001
FT3 (poml/L)	5.33 ± 0.65	4.59 ± 0.88^a^	−0.80 (−1.25 to −0.26)	5.25 ± 0.62	4.14 ± 0.86^a^	−0.75 (−1.40 to −0.62)	0.194
FT4 (pmol/L)	16.1 ± 1.8	17.8 ± 2.6	0.3 (−0.1 to 2.2)	16.5 ± 2.2	16.5 ± 2.3	−0.1 (−1.3 to 1.4)	0.172
FT3/FT4	0.33 (0.29 to 0.35)	0.27 (0.25 to 0.30)^a^	−0.05 (−0.09 to −0.03)	0.32 (0.30 to 0.33)	0.26 (0.24 to 0.28)^a^	−0.05 (−0.08 to −0.03)	0.875
TT4RI	28.5 ± 9.2	27.4 ± 9.1	−3.3 (−9.1 to 7.5)	53.1 ± 8.4	38.8 ± 14.0^a^	−10.4 (−23.2 to −5.5)	0.000

^a^Significantly different at *P* < 0.01 vs baseline; ^b^Significantly different at *P* < 0.05 vs baseline.

adipo-IR, adipose tissue insulin resistance; Fasting FFA, fasting free fatty acid; FBG, fasting blood glucose; FINS, fasting insulin; FT3, free triiodothyronine; FT4, free thyroxine; HDL-C, high-density lipoprotein cholesterol; HOMA-IR, homeostasis model assessment of insulin resistance; LDL-C, low-density lipoprotein cholesterol; TC, total cholesterol; TSH, thyroid-stimulating hormone; TT4RI, thyrotroph T4 resistance index.

## Discussion

This study showed that participants from lean to obesity, metabolism disorders were increased, and adipo-IR, TSH, FT3, FT3/FT4 and TT4RI were also significantly increased. Adipo-IR was significantly correlated with BMI, TSH, FT3 and FT3/FT4. After 6 months of bariatric surgery, BMI, dyslipidemia, TG, A1C, FBG, FINS, fasting FFA, HOMA-IR, adipo-IR, TSH, FT3, FT3/FT4 and TT4RI were significantly declined, and HDL-C level was significantly elevated. We also observed that bariatric surgery notably improved glucose homeostasis and insulin sensitivity, reflected in the improvement of OGTT, insulin release, DI, EISI (ΔI_30_/ΔG_30_) and HOMA-IR. Interestingly, the decrease in BMI after bariatric surgery was significantly associated with the decreases in adipo-IR and TSH. To further study the association between TSH and adipo-IR, we stratified preoperative TSH and found that the adipo-IR in the higher TSH group decreased more remarkably than in the lower TSH group, although weight loss had no difference between the two groups.

Adipose tissue is an endocrine organ and plays a core role in whole-body metabolism. Adipocytes release multiple anti-inflammatory and pro-inflammatory cytokines. A low degree of inflammation is a link between IR and obesity. Studies showed that elevated proinflammatory cytokines TNF-α and IL-6 impaired insulin receptor substrate-1 (IRS1) and insulin receptor phosphorylation by down-regulating PPARγ receptors and up-regulating SOCS3 proteins, resulting in IR (15, 16). IR induced by obesity could lead to an elevated inflammatory state by disturbing the anti-inflammatory role of insulin (17, 18). Bariatric surgery could alleviate the chronic inflammatory state of obesity. Some studies have shown that concentrations of TNF-α, IL-6 and CRP in obese individuals decreased significantly after bariatric surgery (19, 20, 21). Recent studies have reported that HOMA-IR and adipo-IR significantly decreased after bariatric surgery, which was compatible with the results of this study (5, 6). For the first time, we found that the reduction in adipo-IR after bariatric surgery was significantly correlated with the reduction in BMI and TSH, and the decrease in the adipo-IR level in the higher TSH group was more remarkable than in the lower TSH group. The improvement of inflammation after bariatric surgery might be one reason for the decrease of adipo-IR and TSH.

Many studies have demonstrated increased TSH concentrations in morbidly obese patients (22, 23, 24). However, previous studies showed different TSH variation results after bariatric surgery (11, 14, 25, 26). In our study, we found a linear correlation between BMI, TSH and adipo-IR at baseline, which was consistent with the relationship among changes in BMI, TSH and adipo-IR before and after bariatric surgery. Moreover, the decline in adipo-IR in the higher TSH group was more obvious than the lower TSH group 6 months after surgery. However, the potential mechanism for the change in TSH after bariatric surgery remains unclear. Growing evidence showed that inflammatory markers, such as CRP, IL-6 and TNF-α, are elevated in subclinical hypothyroidism patients (27), which could be reversed by bariatric surgery (19, 20). In the study by Shen Qu *et al.*, the results revealed a correlation between the decrease in TSH and decrease in CRP, IL-6 and TNF-α in subclinical hypothyroidism patients; a correlation between the reduction in TSH and reduction in TNF-α in euthyroid patients, suggesting that the amelioration of inflammatory state might contribute to the alteration of TSH after bariatric surgery (21). The declined leptin concentration after bariatric surgery has been proved in some studies (28, 29). Leptin is mainly expressed in adipose tissue, regulates fat mass and body weight and exerts an important role in energy expenditure (30). Leptin could keep thyrotropin-releasing hormone (TRH) expression via the JAK/STAT pathway and then stimulate the production of TSH and thyroid hormones (31). Therefore, the reduction of leptin induced by bariatric surgery could result in the decline of TSH. Interestingly, we found positive relationships between BMI, TSH, FT3 and FT3/FT4 in the context of the negative feedback regulation loop of the thyroid hormone axis. Regularly, if FT3 and FT3/FT4 increase, TSH should decrease; if FT3/FT4 decrease after surgery, TSH should increase. However, this was not the case in our study. The probable reason might be thyroid hormone resistance in severely obese patients (14); in our study, TT4RI was observed to elevate in obese patients and decrease after bariatric surgery, but it was still higher than the regular TT4RI level reported in other studies (14, 32). The exact mechanism needs to be elucidated in further animal studies.

A previous study showed that in white adipose tissue, the thyroid hormone receptor (TRA1) and the TSH receptor (TSHR) of obese individuals were reduced in proportion to the extent of obesity. Weight loss after bariatric surgery, the expression of TRA1 and TSHR was significantly increased in s.c. adipose tissue, with a decrease of serum FT3 and TSH levels (33). In the study by Xia *et al.*, the *TSHR* gene expression was positively associated with the mitochondrial function gene expression (*PPARGC1A*, *SIRT1*, *CISD1*, *ISCA2*, *NRF1*, *NFE2L2*) and fatty acid mobilization gene expression (*ENG1, CAV1*) in both s.c. and visceral adipose tissue. Weight loss induced by bariatric surgery increased s.c. adipose tissue *TSHR* gene expression parallel to increased *PPARGC1A*. These data suggested that adipose tissue TSH might play a character in the maintenance of adipocyte mitochondrial function (10). Mitochondria are the energy centers of adipocytes and participate in several pivotal metabolic functions, including the production of ATP, the synthesis and oxidation of fatty acid and the balance of cell triglyceride (34). TSHR is a G protein-coupled receptor (GPCRs) (35). The activation of TSHR could up-regulate kinases, such as phosphoinositide-3 kinase (PI3K) and P70S6K (36, 37). Crosstalk between cell-surface GPCRs and receptor tyrosine kinases (RTKs) is a pervasive phenomenon (38). The insulin-like growth factor 1 receptor (IGF1R) is an RTK shown to have crosstalk with TSHR in brow fat and orbital fat of patients with thyroid-associated ophthalmopathy (39, 40, 41). Insulin signaling and IGF1 signaling are crucial to adipose tissue development and function (42). With the participation of insulin receptor, insulin gives rise to autophosphorylation, and IRS is recruited and phosphorylated. The IRS proteins then recruit and activate PI3K, which phosphorylates PIP2 to produce PIP3. PIP3 activates PDK1, which phosphorylates Akt at Thr308 (43). Both TSHR and IGF1R could activate PI3K/AKT pathway, regulating insulin resistance.

To sum up, we speculated that weight loss after bariatric surgery, inflammatory status could be reversed, and the expression of TSHR could be increased in adipose tissue, with improved adipocyte mitochondrial function, activated PI3K/AKT pathway alone and with IGF1R crosstalk. Therefore, we speculated that the decline of TSH is one of the most important mechanisms for the decline of adipo-IR after bariatric surgery.

This study has several limitations. First, our research was a retrospective study, gender and age varied in the proportion of lean, overweight and obese groups. Secondly, we did not detect leptin and energy metabolism during the enrollment of participants, and we could not trace the data afterwards. Thirdly, adipo-IR is also dependent on the diet (carbohydrate vs lipids), but we did not control for the ratio of carbohydrate to lipid intake. Fourthly, we found that the elevated TSH may be beneficial to the improvement of FBG, FINS, adipo-IR and TSH after bariatric surgery in non-diabetic obese patients. However, we did not verify the underlying mechanism. Moreover, further experiments are needed to find out the molecular mechanisms of how TSH/TSHR effect adipo-IR.

## Conclusions

In conclusion, obese patients were accompanied by elevated TSH and adipo-IR levels. TSH and adipo-IR levels of morbidly obese patients were significantly reduced 6 months after bariatric surgery; the adipo-IR, FBG, FINS and TSH levels in the higher TSH group decreased more remarkably than in the lower TSH group. Therefore, in obese patients with higher TSH levels within the reference range, metabolic improvement was more obvious after bariatric surgery.

## Supplementary Material

Supplemental Table 1. Correlation between BMI, Adipo-IR, TSH and Clinical Parameters.

Supplemental Table 2. Correlation between the Change in BMI, Adipo-IR, TSH and Clinical Parameters before and after Bariatric Surgery.

## Declaration of interest

The authors declare that there is no conflict of interest that could be perceived as prejudicing the impartiality of the research reported.

## Funding

This work was supported by grants from the Chinese National Natural Science Foundation (No. 81770792) and Beijing Hospitals Authority Clinical Medicine Development of Special Funding Support (ZYL X20 2106) to G W; and the Foundation of Beijing Municipal Science & Technology Commission (No. Z151100004015065) to H M.

## Author contribution statement

Conception or design: G W, J L, H M. Acquisition, analysis or interpretation of data: N B, X S, B Z, L Z, Q W, Y A, X L, Y L, J L, H M, and G W. Drafting the work or revising: N B, J L, G W. Final approval of the manuscript: G W.
