# Supplementary material for: Obese patients with higher TSH levels had an obvious metabolic improvement after bariatric surgery
Source: Endocr Connect. 2021 Sep 15;10(10):1326–36. doi: 10.1530/EC-21-0360 (PMC8558898; doi:10.1530/EC-21-0360)
Supplement: Supplemental Table 1. Correlation between BMI, Adipo-IR, TSH and Clinical Parameters. [file supplementary_table_1.pdf]

Supplemental Table 1. Correlation between BMI, Adipo-IR, TSH and Clinical Parameters.

Variable	BMI		Adipo-IR		TSH	
	r	*P*	*r*	*P*	*r*	*P*
Age	-0.193	0.001	-0.454	0.000	-0.281	0.000
Gender	-0.464	0.000	-0.030	0.623	0.199	0.002
BMI	-	-	0.746	0.000	0.309	0.000
Dyslipidemia	0.118	0.045	0.186	0.002	0.068	0.296
Triglyceride	0.268	0.000	0.316	0.000	0.154	0.017
TC	0.086	0.149	0.171	0.005	0.073	0.259
HDL-C	-0.469	0.000	-0.383	0.000	-0.095	0.144
LDL-C	0.251	0.000	0.305	0.000	0.156	0.016
AIC	0.174	0.006	0.215	0.001	-0.065	0.354
FBG	0.369	0.000	0.431	0.000	0.068	0.296
FINS	0.743	0.000	0.916	0.000	0.371	0.000
Fasting FFA	0.286	0.000	0.527	0.000	0.015	0.814
HOMA-IR	0.720	0.000	0.902	0.000	0.301	0.000
Adipo-IR	0.746	0.000	-	-	0.402	0.000
TSH	0.309	0.000	0.402	0.000	-	-
FT3	0.320	0.000	0.332	0.000	0.111	0.088
FT4	-0.113	0.073	-0.057	0.384	-0.118	0.070
FT3/FT4	0.358	0.000	0.344	0.000	0.263	0.000

TT4RI	0.221	0.001	0.282	0.000	0.936	0.000

BMI, body mass index; TC, total cholesterol; HDL-C, high-density lipoprotein cholesterol; LDL-C, low-density lipoprotein cholesterol; FBG, fasting blood glucose; FINS, fasting insulin; Fasting FFA, fasting free fatty acid; HOMA-IR, homeostasis model assessment of insulin resistance; adipo-IR, adipose tissue insulin resistance; TSH, thyroid-stimulating hormone; FT3, free triiodothyronine; FT4, free thyroxine, TT4RI, thyrotroph T4 resistance index.
