# Supplementary material for: Obese patients with higher TSH levels had an obvious metabolic improvement after bariatric surgery
Source: Endocr Connect. 2021 Sep 15;10(10):1326–36. doi: 10.1530/EC-21-0360 (PMC8558898; doi:10.1530/EC-21-0360)
Supplement: Supplemental Table 2. Correlation between the Change in BMI, Adipo-IR, TSH and Clinical Parameters before and after Bariatric Surgery. [file supplementary_table_2.pdf]

Supplemental Table 2. Correlation between the Change in BMI, Adipo-IR, TSH and Clinical Parameters before and after Bariatric Surgery.

Variable	△BMI		△Adipo-IR		△TSH	
	r	*P*	*r*	*P*	*r*	*P*
△BMI	-	-	0.577	0.000	0.401	0.005
△Dyslipidemia	0.047	0.758	0.267	0.134	-0.142	0.353
△Triglyceride	0.098	0.512	0.032	0.858	0.008	0.960
△TC	0.156	0.295	0.066	0.712	0.032	0.831
△HDL-C	-0.065	0.662	-0.107	0.549	0.116	0.438
△LDL-C	0.117	0.435	0.097	0.585	-0.016	0.917
△AIC	0.109	0.466	0.129	0.467	0.054	0.718
△FBG	0.325	0.026	0.363	0.035	0.119	0.425
△FINS	0.180	0.315	0.169	0.440	0.195	0.286
△Fasting FFA	0.014	0.924	0.043	0.809	-0.029	0.844
△HOMA-IR	0.614	0.004	0.527	0.096	0.370	0.130
△Adipo-IR (	0.577	0.000	-	-	0.536	0.001
△TSH	0.401	0.005	0.536	0.001	-	-
△FT3	0.065	0.662	0.166	0.341	0.235	0.108
△FT4	0.183	0.214	0.243	0.160	0.155	0.292
△FT3/FT4	-0.047	0.753	0.004	0.980	0.088	0.553
△TT4RI	-0.266	0.067	-0.425	0.062	0.933	0.000

BMI, body mass index; TC, total cholesterol; HDL-C, high-density lipoprotein cholesterol; LDL-C,

low-density lipoprotein cholesterol; FBG, fasting blood glucose; FINS, fasting insulin; Fasting FFA, fasting free fatty acid; HOMA-IR, homeostasis model assessment of insulin resistance; adipo-IR, adipose tissue insulin resistance; TSH, thyroid-stimulating hormone; FT3, free triiodothyronine; FT4, free thyroxine; TT4RI, thyrotroph T4 resistance index.
